# The Speech, Spatial and Qualities of Hearing Scale (SSQ)

**DOI:** 10.1080/14992020400050014

**Published:** 2004-02

**Authors:** Stuart Gatehouse, William Noble

**Affiliations:** * MRC Institute of Hearing Research, Glasgow, UK; † University of New England, Armidale, NSW, Australia

**Keywords:** Disability, Handicap, Speech hearing, Spatial hearing

## Abstract

The Speech, Spatial and Qualities of Hearing Scale (SSQ) is designed to measure a range of hearing disabilities across several domains. Particular attention is given to hearing speech in a variety of competing contexts, and to the directional, distance and movement components of spatial hearing. In addition, the abilities both to segregate sounds and to attend to simultaneous speech streams are assessed, reflecting the reality of hearing in the everyday world. Qualities of hearing experience include ease of listening, and the naturalness, clarity and identifiability of different speakers, different musical pieces and instruments, and different everyday sounds. Application of the SSQ to 153 new clinic clients prior to hearing aid fitting showed that the greatest difficulty was experienced with simultaneous speech streams, ease of listening, listening in groups and in noise, and judging distance and movement. SSQ ratings were compared with an independent measure of handicap. After differences in hearing level were controlled for, it was found that identification, attention and effort problems, as well as spatial hearing problems, feature prominently in the disability–handicap relationship, along with certain features of speech hearing. The results implicate aspects of temporal and spatial dynamics of hearing disability in the experience of handicap. The SSQ shows promise as an instrument for evaluating interventions of various kinds, particularly (but not exclusively) those that implicate binaural function.

## Introduction

What are the disabling effects of a deficit in hearing, and what are the relative impacts of those disabilities on the degree of handicap experienced by the person whose hearing is impaired? These are the questions we offer answers to in the present paper, at least with reference to adult clients of a typical audiology clinic. The universal view in the rehabilitative enterprise is that reduced hearing for speech is the primary issue, such that if this function can be properly addressed, the handicaps of the person with diminishing hearing ability will be substantially reduced. Improving the audibility of speech, including compensating for reduced dynamic range and suppression of competing noise, is the goal of hearing aid designers, and this goal has first priority in audiological principle and practice.

The auditory system serves other functions besides speech hearing, such as the localization of sounds. The importance of this aspect of hearing has been argued for within the context of audiological rehabilitation (e.g. Byrne & Noble, 1998). In addition, it is evident that people use hearing for identifying and distinguishing between audible events, both in daily listening contexts and in recreational ones, such as listening to music. Hearing has been characterized by Bregman (1990), using the concept of ‘auditory scene analysis’. This construes the task of hearing as one of partitioning (‘parsing’) the various overlapping sound streams that a listener is typically confronted with, in order to recover a coherent array of signals.

Here we elaborate on hearing as ‘scenic analysis’, and the implications we see for disability assessment. Sounds occur around us virtually all the time, deriving from multiple sources at multiple locations at varying points in time. When a sound is salient, a listener shifts attention, eyes and head towards the source, and listens carefully. We comprehend the sound, and may participate in communication, principally in the form of dialogue. The auditory system, and deficits in its function, are integral to the cascade of hearing, listening, comprehending and communicating (cf., Noble, 1983). Traditional audiological research pays little attention to the ecological complexities of human communication. Performance measures in the laboratory or clinic usually test the segmental intelligibility of a single voice, whose spatial position and spectral /temporal characteristics are static and predictable, in a single noise (usually steady state or at best speech-like babble), which is again static and predictable. In elderly listeners with sensorineural hearing loss, the measured audibility of an earphone-delivered speech signal, together with measures of frequency and temporal resolution, have high predictive leverage on the segmental intelligibility of a single talker in background noise.

Such measures are much less predictive for listening in real rooms with a variety of reverberation characteristics, containing multiple sound sources, some of which are talkers, and some non-speech. In these real contexts, listeners must locate, identify, attend to and switch attention between signals, so as to maintain communicative competence and a sense of connection with their surroundings. Even though sensorineural hearing loss is typically cochlear in origin, the interaction between sensory and cognitive aspects of hearing must exert a material influence on how listeners function in real environments. This interaction will bear on the degree of disability experienced, and the benefits of intervention.

In the self-report domain, it is possible to represent a richer set of communication environments, but traditionally this approach still ignores the three-dimensional and temporally dynamic aspects of the auditory world. The great majority of items that contribute to scales such as the Abbreviated Profile of Hearing Aid Benefit (Cox & Alexander, 1995) assume or imply a listening circumstance where the target voice is predictable in both space and time.

In contrast, when we attempt to identify the elements and tasks in a perceptually more demanding acoustical environment, several additional factors come into play. When a listener has to attend to one conversation in the presence of several similar speakers, selective attention to that conversation occurs via object or stream segregation. But the listener also needs to be ready to attend to new conversations in this sort of setting, requiring monitoring of the auditory environment for the occurrence of new input streams, to assess their salience, to locate them, and, if necessary, to switch attention to a different stream. A strategy of exclusive selective attention would not be appropriate.

Switching attention from one conversation to another means that multiple ongoing streams must be simultaneously monitored to allow salience to be updated and attention refocused as appropriate. In this circumstance, there may or may not be acoustical changes; rather, the content or meaning of the message will influence attentional re-allocation. Finally, listeners will decide or need to engage and disengage in conversations as talkers approach or recede from their spatial location in the auditory world, thereby requiring an ability to monitor the spatial and temporal dynamics of multiple streams and to track their acoustical and content properties. In all of these activities, whatever other listener features are called upon, binaural hearing is powerfully implicated.

The foregoing considerations lead us to suggest that traditional self-report methods of assessing the components and degrees of auditory disability omit or at least underestimate the contributions of functions and ways in which deficits in hearing might lead to increased difficulties for listeners. Our objective in generating the present inventory has been to assemble items that tap into the range of abilities and capacities that we identify as important, and then to investigate the ways in which and extent to which any reported deficits in these capacities relate to the experience of handicap.

In considering the objective of disability assessment, we have identified three general domains, namely speech, spatial and ‘other qualities’ of hearing. Part of the background has been provided by Noble et al (1995), who devised a questionnaire focusing on spatial and speech hearing. In the present project, further speech-hearing contexts have been itemized, reflecting the scenarios described above, and the category of spatial hearing has been expanded to include coverage of movement perception and discrimination. In addition, items have been composed addressing other functions and qualities of hearing, again consonant with the above scenarios, namely, signal segregation, identification/recognition, clarity and naturalness, and ease of listening.

In the present paper, we report on the pattern of responses using the resulting questionnaire with a group of hearing clinic clients, how that response pattern relates to self-rating on social and emotional handicaps, and how the items comprising the questionnaire relate to each other. The new questionnaire is entitled the Speech, Spatial and Qualities of Hearing Scale; SSQ for short. It currently comprises 14 scored items on speech hearing, 17 on spatial hearing, and 18 on the other functions and qualities listed above. When it is applied to aided listening, additional items are available.

The initial application of the SSQ addresses the two-part question posed at the start of this paper—what is disabling about hearing impairment, and how do those disabilities determine the experience of handicap? Our interest has been in observing the incidence and degree of disabilities across the domains represented by the SSQ in a sample of people newly referred for audiological services. We also observe the connections between those disabilities and an independent measure of handicap that addresses restrictions on participation as well as negative emotional effects due to hearing difficulties. Finally, we examine interrelationships among the items of the scale to observe how different functions of hearing covary with other ones, and equally, what functions appear to be independent.

The SSQ focuses on several hearing functions that are presumed to be served to advantage by the binaural system. Indeed, issues of binaural interaction and summation gave part of the impetus to the project in the first place. The binaural issue is included as part of the analysis described in this paper: we examine it more particularly in the companion paper that follows this one, dealing with the role of interaural asymmetry of hearing loss.

## Methods

### Questionnaire development

The content of the first part of the SSQ, on speech hearing, was designed to cover an extensive but realistic range of speech-hearing contexts, and of substantial variation in assumed difficulty. The items covered conditions of competing sounds, the visibility of other talkers, the number of people involved in a conversation, and differences in background conditions (quiet, constant noise, reverberation, many other voices). Some of these items resemble those in the Noble et al (1995) questionnaire. Several of them address functions that are likely to implicate the binaural system; items were also drawn up with particular binaural emphasis, covering selective, divided and rapidly shifting attention—ignoring one voice while attending to another, following two speech streams at the same time, or following conversation that switches quickly from one person to another. We recognize that although these latter items, on the face of it, address speech hearing, they may well cluster with items on ease of listening, in the third (‘other qualities’) section of the SSQ. Indeed, more broadly, the set of qualities items includes domains of inquiry (e.g. segregation and clarity of signals) that follow from the abilities assessed in the first two parts of the scale.

The second section of the SSQ, on spatial hearing, addresses ‘classical’ components of that domain: directional and distance judgments (although the latter are often under-represented in many inventories). We extended our inquiries to cover the discrimination of movement, and in analysis and discussion we consider spatial hearing in terms of three components: direction, distance, and movement. Spatial hearing can be thought of in terms of stationary events, and this, in part, reflects reality. Many audible occurrences whose direction or distance is salient for a listener are stationary, but the layout of the ‘auditory scene’ is also dynamic. Objects and people move, and on paths that will have implications for a listener—for example, approaching/retreating versus moving at a tangent relative to oneself.

Spatial dynamics are specified by temporal dynamics, e.g. by changes in loudness. Indeed, temporal dynamics are implicated in many everyday auditory scenes: patterns of change in loudness mark features such as change in vocal emphasis or change of emotional tone. As outlined in the Introduction, we see this as part of a neglected yet important element not only of spatial hearing but of hearing in general. The spatial section of the SSQ also included reference to ‘externality’ of sounds—do they seem ‘out there’ or ‘in the head’? This item refers more to listening with hearing aids, where ear moulds lead to occlusion, and hence is likely to be relevant in future use of the SSQ post-fitting. Items were also composed to inquire about sounds being nearer or further away than expected.

The third section of the SSQ, on ‘other qualities’, contains items addressed to the issues of segregation of sounds, recognition, clarity/naturalness, and listening effort. On segregation, the questions were about being able to experience simultaneous sounds as separate entities rather than mixed together. In items on identifying or recognizing different sounds, examples of music and speech were used (e.g. ‘Do you find it easy to recognize different people you know by the sound of each one’s voice?’). Items on clarity and naturalness inquired about everyday sounds, others’ voices (including detection of mood from another’s voice), and the naturalness of one’s own voice. Ease of listening items asked about how effortful it was to follow a conversation or ignore interfering noise. We borrowed the sense of some of these topics from existing measures: recognizing sounds is one component of the Amsterdam Inventory (Kramer et al, 1995); ease of listening features in the Profile of Hearing Aid Performance (Cox & Gilmore, 1990).

We have stressed the issue of binaural hearing. Nonetheless, there are contexts in which listening relies more on one ear (or the other) rather than two. In addition to an item on telephone listening in the speech section of the SSQ (which we assume involves single-ear listening), we selected the cases of being the driver in a car and hearing what the passenger next to you is saying, and of hearing the driver alongside you while being the passenger. These two items were located in the ‘qualities’ section of the questionnaire, but we recognize that they might turn out to relate to items on speech hearing.

Prototype versions of the SSQ were piloted with one or other of the authors as interviewer, and the other as observer. We enlisted the help of a range of hearing clinic outpatients at the Glasgow Royal Infirmary for this purpose. The wording of items was adjusted where it was clear that the intended meaning was ambiguous or confusing. Wording was also adjusted so that, as far as possible, audibility was not in question for any respondent. New items were added to the ‘qualities’ section when it was judged from interviews that topics could usefully be given more emphasis. Several iterations were undertaken by these means.

### Procedure

The version of the SSQ that was used in all subsequent interviews is shown in Appendixes 1 and 2. (Appendix 1 shows a sample SSQ item with the scoring ruler described below. Appendix 2 gives the text of each item and the endpoints on the scoring ruler.) These interviews were conducted by two audiologists, experienced in administering such instruments, and given specific training by the authors to ensure that all questions would be understood and meaningfully answered. The interview approach was preferred over self-administration, so as to ensure that the precise contexts were understood by each respondent, and also because an interview setting allows elaboration where an item’s meaning seems to have been misconstrued.

The scoring scheme for each item was identical, using the ruler representation shown in Appendix 1. The left-hand end of the ruler represented complete inability, or complete absence of a quality (or, in the case of ‘effort’ items, complete need for such effort). The right-hand end represented complete ability, or complete presence of a quality (or complete absence of need for effort). Respondents rated themselves on each item with a score out of 10: thus, higher scores always reflect greater ability (less disability). The anchoring terms were varied on items where the standard terms (‘not at all’ and ‘perfectly’) would be inappropriate for the sense of the item, while still retaining the left-to-right negative-to-positive direction of responding. Such variant anchor terms can be seen against several items in Appendix 2. Consistent placement of ‘less able’ responses at the left-hand extreme of the scoring ruler, and ‘more able’ at the right, is preferred to an approach that randomizes the scaling direction. Evidence from a number of sources (review: Noble, 1998, p. 26) shows that reversal of scaling direction simply creates confusion for respondents, who, in the context of the typical hearing clinic, are predominantly elderly.

None of the respondents had used a hearing aid, so all items were rated unaided (additional items in section 3, about hearing aid or implant use, were omitted from the interview). There was provision for any item to be voted as inapplicable or for the sound in question to be judged inaudible, although we tried to include only settings where audibility would not be the primary issue. One of the motivations for an interview format was to achieve dissociation from simple audibility. Very few items failed to elicit a meaningful response from all participants. The most notable example of an item not being applicable was the case of being the driver in a car. Only 37 of 153 respondents reported currently engaging in car driving, reflecting the particular social and demographic features of our sample population.

Prior to visiting the clinic for an initial appointment, the cohort of outpatients was sent the 12-item, five-point-scaled handicap questionnaire, and asked to complete it in advance of that appointment. The 12 questions are listed in Appendix 3. This questionnaire was devised to provide a measure of personal and social effects—emotional distress and discomfort, social withdrawal, and general restriction on participation. It was derived in part from items in the Hearing Disabilities and Handicaps Scale (Hétu et al, 1994), and from items in an unpublished general health scale (the Glasgow Health Status Inventory), with adjustment of wording to ask specifically about effects of hearing. The latter are state versions of the direct-difference questions in the Glasgow Benefit Inventory (Robinson et al, 1996). Each of the items included in the handicap questionnaire has demonstrated appropriate leverage in previous applications. We wanted the scale to focus on consequences for the personal and social self, and to be quite removed from the disability domain, in two ways: (1) in terms of the content of the handicap scale items; and (2) in terms of the occasions for completing the handicap scale and the SSQ. The content of each handicap item is arranged to be independent of any particular listening circumstance or capability. Also, by having potential participants complete the handicap scale for themselves, in advance of visiting the clinic, and by their having no prior awareness of an invitation to engage in the SSQ interview, we hoped to make the assessments as independent from each other as possible. We argue that this is a better procedure than one where respondents give a handicap rating for a given scenario immediately following each disability rating, the approach used in a study with the Amsterdam Inventory (Kramer et al, 1998).

Handicap scale items were scored using five equal intervals from (i) to (v) (see Appendix 3), averaged to give an individual’s global handicap score, and then scaled to have a possible range from 0 to 100. Higher scores equal greater handicap (thus we expect negative correlations with SSQ scores). A preliminary factor analysis did not reveal separate factor structures in the handicap domain; hence the use of a simple unweighted average. (We do, though, acknowledge that a more detailed assessment of handicap will yield a more complex structure, and hence a more complex set of relationships with indices in the disability domain.) Those outpatients who had completed the handicap scale by the time of visiting the clinic, and who consented to be interviewed, were also asked for permission to transcribe the results of their clinic audiogram. Greater values for hearing thresholds imply greater impairment, and we also expect negative correlations between the audiogram and SSQ scores.

## Results

### Descriptive statistics

Scores on some SSQ items were not normally distributed, and non-parametric statistics have been used to correlate outcomes across the various measurements. We use means and standard deviations to present the descriptive statistics within each test.

There were 153 people in the sample (80 females, 73 males), of average age 71 years (SD 8.1). The better-ear average (BEA) hearing threshold over 500, 1000, 2000 and 4000 kHz was 38.8 dB (SD 15.5), where, for each person, better represents the ear with lower hearing level averaged over the frequencies 500, 1000, 2000 and 4000 Hz; the worse-ear average (WEA) was 52.7 dB (SD 24.4). The average score over all items of the SSQ was 5.5 (SD 1.9), and the average handicap score was 49.0 (SD 23.9). The correlation (Spearman’s rho) between BEA and WEA was 0.72. The correlation between BEA and SSQ average was –0.51; that between WEA and SSQ average was –0.52. The correlation between SSQ average and handicap score was –0.61. The correlation between handicap score and BEA was 0.12 (WEA, 0.13). Thus, although there are good correlations between impairment (threshold) and disability (SSQ), and between disability and handicap, the link between impairment and handicap is slight. This conforms with previous observations (e.g. Hétu et al, 1994).

Table 1 shows mean scores (and SDs) on each item in the SSQ, section by section, ordered from lowest to highest scores within each section. The three subscales represent the unweighted averages of the scale items, with the item on internalization omitted from the Spatial subscale, and the item about ability when the driver of a car omitted from the Qualities subscale (as noted elsewhere, these two items differ in important respects from others—hence the exclusion).

Scores on the items in the speech section were generally lower (greater disability) than in the other sections, and cover a considerable range: from 7.1 (out of a maximum 10) to 2.5. The arrangement of scores across items is orderly, and follows expectations. Thus, the highest ratings were for one-to-one conversations in quiet or on the telephone. The next highest were for one-to-one conversations, even in competitive conditions, and group conversations in quiet where all speakers are visible. The items providing lowest ratings were: divided-attention contexts (trying to follow two speech streams simultaneously); group conversations in noise or where not every speaker is visible; echoic environments; and following a group conversation without missing the start of the next speaker.

Average scores in the second section of the SSQ, on spatial hearing, range from 7.5 to 4.2, with items on distance and lateral-movement discrimination showing low ratings. In general, respondents rated directional ability higher than either distance or movement, but this outcome is not uniform. Some of the directional items may be tempered by other factors. Locating the direction of a lawnmower or a vehicle in the street was rated lower than locating a barking dog, a door being slammed, or a speaker in a conversation. The former items have a movement component and, given the lower ratings for movement discrimination, this may have influenced the scores. The item on sounds turning out to be closer than expected had a lower score than the one on sounds turning out to be further away; that is, the experience of them turning out to be closer is more common. With reduced hearing, many sounds will be experienced as quiet, and hence assumed to be further away, but will turn out to be closer.

The scores on items in the third section range from 8.3 to 3.7. The items on ease of listening had the lowest scores, and those on recognizing familiar voices and music had the highest. Judging someone’s mood from their voice, and the naturalness of one’s own voice, were also at the high rating end. The ability to segregate one sound from another was rated between 6 and 7, as were ratings of naturalness and clarity of voices. Naturalness of other everyday sounds, and of music, had slightly higher ratings than this. The items about listening when driving versus when being a passenger in a car had respectively low and moderate ratings.

### Correlation with handicap

We have adopted an incremental approach to analysing the links between disability and handicap, relying on direct followed by partial correlation. In the absence of a standard non-parametric partial correlation procedure, the ranks used in calculating Spearman’s rho have also been used for calculating Pearson partial correlation coefficients. In Table 2, the SSQ items are again shown, but this time ordered, within sections, from highest to lowest direct correlation with handicap. There is no evident linearity between these item orders within each section and the ones shown in Table 1, although, in the speech section, items with low ratings (leftmost numeric column) tend to show higher correlations with handicap. More obviously, no single section (or subset of items within a section) of the SSQ dominates in the association with handicap score, indicating that the range of functions addressed by the SSQ overall feeds into the handicap experience. In saying this, we recognize that other factors, such as cognitive and attentional abilities, which are not assessed in this phase of the project, may be found to moderate aspects of this relationship.

The point about the broad range of connection between disability and handicap calls for further examination to learn if and how the links are leveraged by features of impairment. Two key features in the present context are: auditory *sensitivity* and interaural *asymmetry*. We would expect that a scale designed to assess a variety of hearing functions would show intelligible relations with measured sensitivity, and that a scale focused on binaural functions would show intelligible responsiveness to departures from normal binaurality. Sensitivity is reflected in degree of impairment in the better ear, and asymmetry by degree of difference between BEA and WEA, controlling for BEA (the latter to ensure that any asymmetry effect is isolated from the influence of BEA).

In numeric column 2 of Table 2, the correlations between each SSQ item and BEA are shown. In general, these show that greater impairment is associated with greater disability across the range of SSQ items. Exceptions are as follows: in the speech section, talking on the telephone; in the spatial section, experiencing sounds as further away or closer than expected (and internalization of sounds); and within the other qualities section, understanding when driving or being a passenger in a car. Thus, sensitivity impinges on the functions assessed in the SSQ—as would be expected. It would be problematic if questions on disability were not influenced by impairment. Thus, there is reason to assume that the disability–handicap relationship might be moderated by this factor.

The factor of asymmetry (numeric column 3) does not contribute significantly to this picture for most speech-hearing contexts, although the partial correlations are generally in the direction of showing a minor further effect on disability. Notably different are items involving the telephone. In the case of hearing on the telephone (presumably using the better ear) while trying to follow another conversation, greater asymmetry, independently of BEA, is significantly associated with greater disability. However, in the case of trying to use the telephone as such, greater asymmetry is associated with less disability (positive correlation), presumably because potentially distracting sound is less audible in the non-telephone ear.

The picture is quite different in the case of spatial items. Here, asymmetry plays a significant role independently of sensitivity over all the dimensions (although not on every item) included in this section of the scale. Finally, some of the other qualities items are affected by the factor of symmetry, especially those to do with naturalness and clarity of sounds, signal segregation, and effort required in conversation. The overall impression from the analysis to this point is that audibility is a critical influence driving disability across all the domains of interest, and that asymmetry has a critical additional role in several of those domains.

Thus, in the final step to look at the connections between disabilities and handicap, control for the the above two factors is appropriate, and achieved by partialling both BEA and WEA (Table 2, rightmost column). For the most part, the orders of the resulting partial correlations are not materially disturbed relative to the original ones (numeric column 4). More particularly, substantial relationships remain between disability and handicap. We consider each section of the SSQ in turn.

#### Speech

The experience of handicap is influenced most by contexts calling for divided or rapidly shifting attention: following two conversations at once; not missing the start of what the next speaker says; engaging with a group of people. Handicap is also influenced, though, by difficulty talking with one person in quiet conditions, even though the absolute score for this item is the highest achieved. Presumably, observing that one has difficulty in such favourable conditions reinforces the isolation and emotional distress that characterize the dimensions of handicap assessed in the present study. This is an example of dissociation between degree of difficulty and impact upon handicap. Conversation with one other person in competing conditions is not so influential in the experience of handicap, presumably because part of the problem is attributable to the competing conditions themselves, rather than to one’s own inability. These outcomes suggest that, besides simple auditory sensitivity limitations, broader central auditory attentional and cognitive factors are likely to be involved in the way that speech-hearing ability links to experienced handicap.

#### Space

The experience of handicap is as strongly influenced by various dimensions of spatial hearing as it is by contexts of listening to speech. It appears that the variety of ways in which spatial hearing supports everyday function is involved in that influence. Thus, determining the distance and the paths of movement of other people and the distance of vehicles are prominent in affecting handicap, but so is locating a speaker in a group setting. Locating the direction of a barking dog or a vehicle outdoors, having a sense of sounds being where one expects, are also noticeably in the picture. These outcomes suggest that the reliance placed on spatial hearing, both in monitoring dynamic environmental events and in aiding conversational competence, bears on the handicap experience.

#### Other Qualities

The experience of handicap is also strongly influenced by several of the other qualities assessed in the third section of the scale. Particularly to the forefront are issues of identification, especially of others, their mood; the effort needed to sustain conversation, the clarity and naturalness of sounds, and distinctiveness of different pieces of music. We note that the ability to understand when being a passenger in a car comes into the picture here, but observe that this item may more likely belong with speech issues rather than with the domains otherwise represented here. It is plausible to invoke the idea of social competence once again, in seeing how these qualities help to influence the handicap experience. Failures of identification, such as of persons or their mood, may add to a sense of embarrassment and reinforce a desire to avoid social situations, as will the effort needed to engage in conversation. The presence of questions addressing clarity, naturalness and distinctiveness suggests a role for suprathreshold distortion in the handicap experience.

### Intercorrelations

A standard approach to study interrelationships between items and the ways in which they group is to apply some form of factor analysis. Such an approach can be informative, although it should not replace systematic observation. In the present context, using 153 observations on 49 independent variables is likely to lead to unstable solutions; about 500 observations would be required. Preliminary analyses confirm this lack of stability. Although factor analyses within each section are viable, given the interrelationships between items in different sections and their transferability between sections, they are not informative. Thus we restrict our analytical approach to systematic observation.

Almost all SSQ items are positively intercorrelated; we will note later one or two that show little or no relation with other items. The first analysis is of the pattern of positive interrelationships, made by reference to the more prominent ones (≥0.50), all of which are statistically significant ( *p* < 0.01). The analysis is done twice: once using the direct correlations between items, and then with control for BEA and WEA. This is consistent with the previous analysis: it allows observation of how the various disability domains of the scale relate to each other directly, and again when the underlying factors of audibility and asymmetry are controlled for.

The correlations have been grouped into three bands: 0.50–0.59, 0.60–0.69, and 0.70 and above. To appreciate the pattern of association, the distribution of each item’s correlations across the three subsections of the SSQ are shown in Tables 3 (direct) and 4 (partials). The other SSQ items with which each SSQ item is correlated are identified by number; shorthand versions of all items are given in the tables; full-text versions are given in Appendix 2. The extent of correlation is expressed by the typeface of the item number: correlation values in the 0.50–0.59 band are shown in plain font, those in the 0.60–0.69 band are in bold, and those in the 0.70 and above band are in bold underlined. We consider each section in turn.

#### Speech

As can be seen in Table 3, although almost all SSQ items are intercorrelated, the strongest links are typically among items comprising each section. A feature of speech items that address divided or rapidly shifting attention (items 1, 10, 12, 14) is the sign of extensive links with spatial and other qualities items. This is also true for items 2, 5, 8 and 9, but, as noted later, for those last four items this pattern changes substantially under partial correlation analysis.

The other items in the first section of the SSQ are primarily related to each other, with secondary links in some cases to items in section 2 (mainly distance and movement) and 3 (mainly on naturalness and ease of listening). These other items in the speech section may be distinct from items 1, 10, 12 and 14 in that they refer specifically and exclusively to hearing in a conversation, whereas items 1, 10, 12 and 14 bring in issues of rapid attention-switching and divided attention. The pattern among items 1, 10, 12 and 14 supports the view that attentional capacity may function somewhat independently of sheer speech-hearing ability.

#### Space

As regards the spatial hearing section, the most striking feature is the dominant position of the items on distance and movement. Not only are the movement items strongly intercorrelated, but they also connect in varying degrees of strength with all the other spatial items. The outcome offers a picture of spatial hearing as an integrated set of static and dynamic functions. Furthermore, and to reiterate what was noted in the preceding section, elements of the directional, distance and movement components relate to a range of speech-hearing contexts.

Several spatial items show strong associations with a range of ‘other qualities’ items. The spatial item on telling whether a speaker is to one side or another shows extensive association with all the qualities represented in the third section of the scale. The items on movement towards or away from the listener and on sounds being in their expected location show a similar pattern, with particular emphasis on clarity, naturalness and degree of effort involved in conversation. The movement and expected location items are also associated with the ability to judge mood from voice. It is not certain what contributes to discrimination of mood, but it is feasible, as suggested earlier, that loudness modulations are informative both about change of location and about vocal mood.

#### Other Qualities

The items in this section behave somewhat like the spatial ones, clustering strongly with each other, and being generally dominated by items on segregation, clarity and naturalness.

### Partial intercorrelations

The picture emerging from the previous analysis is not radically changed when inter-item correlations are calculated, controlling for the audiometric factors of audibility and asymmetry. The major effect, as can be seen in Table 4, is to brush away or reduce the prominence of certain components of the previous pattern, while leaving others relatively intact. This permits the conclusion to be drawn that certain of the inter-item relationships are more influenced than others by the audiometric factors in question. The related effect is to show the three domains of the SSQ as relatively more autonomous, relatively less joined up with each other, with some noticeable exceptions.

#### Speech

The clustering of items within this section remains largely unchanged, with the exception of item 2 (one-to-one conversation in quiet), whose extensive pattern of links within and beyond the speech section is now very much reduced. This indicates that the factor of audibility in particular is what drives the relationship between this item and all the others, which makes sense, as the context is one in which audibility will be the key feature.

The other noticeable effects are with regard to associations between certain speech items and those in other sections of the scale. The extensive links between speech items 1, 10, 12 and 14 and spatial (especially distance and movement) items remain, although those observed in the first analysis between speech items 2, 5, 8 and 9 and spatial items shrink substantially. Thus, whereas the connections in the latter cases rest on the underlying factors of audibility (and asymmetry), the links in the case of items 1, 10, 12 and 14 implicate auditory, and perhaps also non-auditory, factors related to attentional deployment. As earlier noted, speech items 1, 10, 12 and 14 address divided and/or rapidly shifting attention. Presumably, to the extent that the spatial layout of the auditory scene is accessible to a listener, this supports a capacity to monitor signals simultaneously at different or changing locations within that scene. Connected to this point, there are moderate-to-strong links between directional and movement items and one or more of the segregation items in section 3 of the scale.

#### Spatial

The strong internal connections observed in the first analysis remain intact. Several of the links between directional and ‘qualities’ items observed earlier are now much reduced, but those between spatial item 3 (locating a speaker to one’s left or right) and qualities of segregation, clearness and naturalness remain. This is also the case for the movement items and the general item about sounds being where one expects (item 17).

#### Other Qualities

Most of the internal links remain, although certain items (item 7, identifying musical instruments) and item 12 (naturalness of own voice) become more isolated, suggesting that audibility is an influential factor in these cases. However, the qualities of segregation (items 1–3) continue to be associated with items on clarity and naturalness (items 8–11). Presumably, sounds that are heard as separate from each other are heard as clear and natural. The clarity/naturalness item group and the item on judging mood from voice (item 13) form the more dominant subset in the third section of the SSQ, in being associated with the qualities of segregation and identification, as well as with each other.

Overall, the items within each section of the SSQ relate to each other in substantial and comprehensible ways. It is evident that the functions of speech hearing, spatial hearing and the further characteristics of hearing experience inquired about in the third section of the scale represent distinct domains. It is telling, however, that certain components of spatial hearing are implicated in speech-hearing contexts that involve divided or rapid switching of attention.

#### Isolates

Within each section, there are more isolated items. Talking on the telephone (speech item 13) appears to be relatively independent of all others. The item on ‘internalizing’ (spatial item 14) shows very little connection with any others in the scale; as noted before, this item is considered more relevant to the hearing-aided context. The items assessing whether sounds are further away or closer than expected (spatial items 15 and 16) appear to be only weakly related to others. The item on ignoring competing sounds (qualities item 19) also appears to be only modestly related to other items in that section. The items on understanding when driving or being a passenger yielded internally inconsistent outcomes.

The results from the car-driving/passenger items prompted us to consider whether the factor of symmetry was interacting with hearing in this particular context (in which the side of the better ear could well be critical in its effect). The issue of symmetry features more broadly, as shown in the disabilities–handicap analysis. We stated in the introduction to that analysis that departures from binaurality, reflected in greater versus lesser degrees of asymmetry, will influence results on an assessment device designed to tap functions assumed to depend on binaural hearing. The consequence of these several points was to look again at the data, dividing the group into those showing symmetrical and asymmetrical hearing. This revealed an interpretable array of outcomes (including clarification on some of the foregoing isolated items), but complex enough to merit separate presentation in the companion paper to this one.

## Discussion

It is evident from the principal findings reported in this paper that novel and significant dimensions of everyday hearing have been illuminated by devising and applying the SSQ. This result is due to the extension of inquiry to more complex and dynamic aspects of hearing capacity. At the same time, in general terms, observations regarding the connections among measures of impairment, disability and handicap are consistent with previous research, so we can be reasonably confident that the novel results are not idiosyncratic. In any case, most of the detail in the findings is interpretable, and it is from analyses of the detail, both between disabilities and handicaps, and within disabilities, that the novel features emerge.

Within the specific domain of speech hearing, the current data show that the items most highly correlated with the experience of handicap are those which tap into aspects of selective attention and switching of attention, items that are not found in any of the traditional hearing disability inventories. Thus, even if discussion is restricted to the domain of speech-hearing disability, the picture of how abilities and disabilities link to handicap will be incomplete and inferior if it is concentrated solely on listening circumstances that are static in space and time. The role of selective attention and its bearing on specific auditory and more general cognitive abilities is one which has not been pursued in previous research in the self-report domain. Our results identify these capacities as important in the links between disability and handicap.

In a similar vein, within the domain of spatial hearing the elements that correlate most highly with the experience of handicap are those to do with the appreciation and discrimination of movement rather than the more static appreciations of direction and distance. Again, accessing those elements of the auditory world which vary in space and time provides a greater insight into the drivers of hearing handicap than the more simple and restricted static elements.

On reflection, it is obvious there are substantial parallels between the detection and discrimination of audible movement and that of visible movement. The visible movement of objects in the environment can be understood in terms of translations at tangents relative to a receptor system, including optical expansions and contractions as a function of whether the movement is towards or away from the observer (Gibson, 1979). Detecting and accurately discriminating among different visible movement trajectories is vital for safe and effective orientation. In exactly the same way (crucially for objects not immediately in view), detecting and discriminating among audible movement trajectories serves the observer’s wellbeing and sense of proper connection with the world. Impairments that compromise this sense of ‘well-connectedness’ are reflected in an understandable increase in the experience of handicap.

Bearing upon that point, it is instructive to note that the sets of correlation coefficients in the spatial hearing domain are similar to, if not greater in magnitude, than those in the speech domain in terms of their relationship to the handicap score. So, although specific complaints about deficits in spatial hearing are rarely reported as the predominant reasons for attendance at management clinics, closer inquiry demonstrates that they are at least as strongly linked with handicap as deficits in the speech-hearing domain.

As well as a wider consideration within any domain, it is important to appreciate the connections between domains to gain a more comprehensive understanding of the ways in which disabilities influence handicap. In the ‘qualities of hearing’ domain, it is possible to identify elements that are associated with the ability to segregate input streams into discrete acoustical entities, with the extent to which the naturalness of sounds is preserved and with the extent to which the ‘fidelity’ of the auditory world is maintained. Clearly, these elements will be related to and to some extent driven by capacities in the speech-hearing and spatial hearing domains, and they do exhibit significant associations with the experience of handicap.

Before concluding, we acknowledge the limitations of our current data. Both the scores on the SSQ items and their relationships to handicap are not necessarily a function solely of audition, but might be influenced by other factors in elderly people with sensorineural hearing loss, e.g. generalized cognitive or attentional deficits. A subsequent stage will be to gather SSQ data from samples of elderly people without hearing impairments to determine their ratings on SSQ items and even how they relate to their experience of handicap (note that respondents may report hearing discomfort handicap although having hearing thresholds within normal limits).

In conclusion, several self-report elements have been revealed in this study that tap into a variety of capacities exhibiting a coherent and interpretable pattern of relationships with aspects of auditory impairment and which act as influences bearing upon the experience of hearing handicap. At present, many of these functions and capacities are simply not measurable in the laboratory on clinical populations. Self-report provides an invaluable insight into the classes and types of functions which influence hearing handicap, and one of the challenges for future research will be to develop performance paradigms that allow the mechanisms and deficits in these functions to be investigated more directly.

## Supplementary Material

SSQ B (pre/post)

SSQ C (comparison)

SSQ original

Appendixes

## Figures and Tables

**Table 1:** Mean and standard deviation of the SSQ item scores ordered within each of the three subscale sections from the lowest (most difficult) to the highest (least difficult) scoring item

	Mean	Standard deviation
Speech-hearing items		
Follow one person speaking and telephone at same time	2.5	1.8
Having conversation with five people in noise—no vision	2.7	2.2
Talk with one person and follow TV	3.0	2.6
Having conversation with five people in noise with vision	3.4	2.3
Follow conversations without missing start of new talker	4.0	2.4
Having conversation in echoic environment	4.0	2.4
Follow one conversation when many people talking	4.3	2.6
Having conversation with five people in quiet with vision	4.5	2.7
Talking with one person in continuous background noise	4.6	2.4
Talking with one person with TV on	4.6	2.7
Ignore interfering voice of same pitch	4.9	2.4
Ignore interfering voice of different pitch	5.0	2.6
Have conversation on telephone	6.8	2.1
Talking with one person in quiet room	7.1	2.4
Spatial hearing items		
Judge distance from footsteps or voice	4.2	2.6
Judge distance of vehicle	4.5	2.7
Locate lawnmower	4.6	2.7
Identify lateral movement of vehicle	4.8	2.7
Locate vehicle from footpath	4.9	2.8
Identify lateral movement from voice or footsteps	5.0	2.7
Identify whether a vehicle is approaching or receding	5.3	2.8
Locate above or below on stairwell	5.5	2.8
Identify approach or recede from voice or footsteps	5.6	2.7
Locate speaker round a table	5.6	2.8
Locate dog barking	6.0	2.6
Sounds in expected location	6.0	2.8
Sounds closer than expected	6.1	2.7
Locate a door slam in unfamiliar house	6.1	2.8
Lateralize a talker to left to right	7.0	2.6
Sounds further than expected	7.3	2.2
Internalization of sounds	7.5	2.3
Qualities of hearing items		
Need to concentrate when listening	3.7	2.8
Effort of conversation	4.0	3.1
Understand when driver of a car	4.6	2.8
Ability to ignore competing sounds	5.3	3.1
Understand when car passenger	5.4	2.7
Sounds appearing jumbled	5.9	3.1
Naturalness of other voices	6.0	2.5
Music and voice as separate objects	6.3	2.7
Clarity of everyday sounds	6.6	2.7
Identify instruments in music	6.6	3.0
Separation of two sounds	6.6	3.0
Naturalness of everyday sounds	7.1	2.8
Naturalness of music	7.2	2.6
Judging mood by voice	7.5	2.5
Distinguish different sounds	7.5	2.7
Naturalness of own voice	7.7	2.8
Identify different people by voice	7.8	2.0
Distinguish familiar music	8.3	1.9

**Table 2:** Mean SSQ item scores and rank correlations between the SSQ item and (1) better-ear average, (2) asymmetry controlling for better-ear average, (3) handicap score, and (4) handicap score controlling for better-ear and worse-ear average; the table is ordered within each of the three subscales from highest to lowest correlation with handicap score

	Rank correlation between the SSQ items and:				
	Mean	BEA	Asymmetry controlling BEA	Handicap score	Handicap score controlling BEA and WEA
Speech-hearing items						
Follow one person speaking and telephone at same time	2.5	−0.41**	−0.23**	−0.64**	−0.52**
Follow conversations without missing start of new talker	4.0	−0.48**	−0.07	−0.61**	−0.53**
Talk with one person and follow TV	3.0	−0.40**	−0.02	−0.56**	−0.46**
Having conversation with five people in noise with vision	3.4	−0.21**	−0.11	−0.52**	−0.42**
Having conversation with five people in noise—no vision	2.7	−0.20*	−0.09	−0.49**	−0.33**
Ignore interfering voice of different pitch	5.0	−0.49**	−0.10	−0.48**	−0.30**
Ignore interfering voice of same pitch	4.9	−0.44**	−0.08	−0.45**	−0.25**
Talking with one person with TV on	4.6	−0.46**	−0.13	−0.44**	−0.48**
Talking with one person in quiet room	7.1	−0.49**	−0.14	−0.43**	−0.41**
Follow one conversation when many people talking	4.3	−0.35**	−0.01	−0.41**	−0.26**
Having conversation with five people in quiet with vision	4.5	−0.27**	−0.04	−0.41**	−0.37**
Have conversation on telephone	6.8	−0.09	0.24**	−0.41**	−0.24**
Talking with one person in continuous background noise	4.6	−0.52**	−0.06	−0.37**	−0.14
Having conversation in echoic environment	4.0	−0.48**	−0.05	−0.32**	−0.15
Spatial hearing items						
Judge distance from footsteps or voice	4.2	−0.51**	−0.20*	−0.58**	−0.50**
Identify whether vehicle is approaching or receding	5.3	−0.47**	−0.14	−0.58**	−0.53**
Identify lateral movement from voice or footsteps	5.0	−0.48**	−0.18*	−0.56**	−0.45**
Locate speaker round a table	5.6	−0.30**	−0.24**	−0.55**	−0.49**
Sounds in expected location	6.0	−0.35**	−0.27**	−0.52**	−0.41**
Identify lateral movement of vehicle	4.8	−0.51**	−0.05	−0.50**	−0.41**
Locate dog barking	6.0	−0.42**	−0.25**	−0.49**	−0.43**
Locate vehicle from footpath	4.9	−0.48**	−0.23**	−0.49**	−0.42**
Judge distance of vehicle	4.5	−0.43**	−0.18*	−0.45**	−0.36**
Locate a door slam in unfamiliar house	6.1	−0.16*	−0.29**	−0.44**	−0.35**
Identify approach or recede from voice or footsteps	5.6	−0.42**	−0.26**	−0.44**	−0.33**
Locate lawnmower	4.6	−0.33**	−0.21*	−0.42**	−0.26**
Lateralize a talker to left to right	7.0	−0.42**	−0.25**	−0.38**	−0.18*
Sounds further than expected	7.3	−0.09	−0.15	−0.32**	−0.26**
Locate above or below on stairwell	5.5	−0.41**	−0.25**	−0.30**	−0.25**
Sounds closer than expected	6.1	−0.03	−0.19*	−0.23**	−0.15
Internalization of sounds	7.5	−0.14	−0.03	0.03	−0.01
Qualities of hearing items					
Effort of conversation	4.0	−0.31**	−0.25**	−0.56**	−0.45**
Identify different people by voice	7.8	−0.30**	−0.07	−0.53**	−0.59**
Need to concentrate when listening	3.7	−0.33**	−0.03	−0.51**	−0.49**
Music and voice as separate objects	6.3	−0.26**	−0.05	−0.50**	−0.34**
Naturalness of other voices	6.0	−0.47**	−0.34**	−0.50**	−0.50**
Understand when car passenger	5.4	−0.16	−0.22**	−0.48**	−0.51**
Judging mood by voice	7.5	−0.31**	−0.13	−0.46**	−0.50**
Clarity of everyday sounds	6.6	−0.45**	−0.45**	−0.45**	−0.40**
Distinguish familiar music	8.3	−0.32**	−0.04	−0.42**	−0.44**
Naturalness of everyday sounds	7.1	−0.27**	−0.49**	−0.42**	−0.38**
Naturalness of music	7.2	−0.30**	−0.15	−0.40**	−0.20*
Sounds appearing jumbled	5.9	−0.39**	−0.29**	−0.39**	−0.23**
Distinguish different sounds	7.5	−0.29**	−0.12	−0.38**	−0.24**
Identify instruments in music	6.6	−0.36**	−0.09	−0.36**	−0.32**
Naturalness of own voice	7.7	−0.31**	−0.32**	−0.35**	−0.32**
Understand when driver of a car	4.6	−0.11	0.14	−0.35**	0.43**
Separation of two sounds	6.6	−0.23**	−0.23**	−0.34**	−0.30**
Ability to ignore competing sounds	5.3	−0.28**	0.04	−0.29**	−0.21**

** Correlation is significant at the 0.01 level (two-tailed).

* Correlation is significant at the 0.05 level (two-tailed).

BEA, better-ear average; WEA, worse-ear average.

**Table 3:** Spearman rank correlations between SSQ items; items correlating are identified by number within three subsections

Item	SSQ section	Items correlated, Speech	Items correlated, Spatial	Items correlated, Other
	**I Speech**			
1	talk 1 TV on	**2** 3 4 5 9 10 **14**	**1** 2 **3** 4 5 6 **7 8 9 10 11 12** **13** 17	1 **2** 3 **4** 5 6 **8 9 10** 11 **13 14 18**
2	talk 1 quiet	**1** 3 8 **9** 10 11 12 14	**3** 8 10 12 **13** 17	1 2 3 **4 5** 6 8 **9 10** 11 **13** 14
3	5 quiet see all	1 2 **4** **6** 7 8 **9** 11 12 14	9	10 13 18
4	5 noise see all	1 **3** **6 7 8 9** 11 **12 14**	3 8 13	1 **2** 3 **10** 13 18
5	talk 1 noise	1 **8 9** 12 14	**3 7** 8 9 **10** 11 12 **13**	**2** 3 5 6 **7** 8 **10** 13
6	5 noise not see all	**3** **4 7** 8 9 11 **12 14**	8	3 10 18
7	talk where echoic	3 **4 6 8** **9** **11** **12** 14	7 8 11 13	2 9 **16**
8	ignore voice same pitch	2 3 **4 5** 6 **7** **9** **11** **12 14**	**3** 7 8 9 10 11 13	2 **8** 9 10 16
9	ignore voice different pitch	1 **2 3 4 5** 6 **7** **8** **11** **12 14**	1 **3** 5 7 8 9 10 11 12 **13** 17	2 3 5 **8** 9 **10** 11 12 13 18
10	talk 1 and follow TV	1 2 **12 14**	**2** 4 6 7 8 **10** 11 **12 13**	14 **16**
11	talk 1 other voices	2 3 4 6 **7** **8** **9** **12**		16
12	not miss speaker start	2 3 **4** 5 **6 7** **8** **9 10 11** **14**	**2** 5 6 **7 8 9 10 11** 12 **13**	9 10 16 18
13	talk on phone			
14	follow 1 and phone	**1** 2 3 **4** 5 6 7 **8 9 10 12**	1 2 3 4 6 **7 8 9 10 11 12 13 17**	1 2 3 4 6 **8 9 10** 11 12 13 14 17 **18**
	**II Spatial**			
1	locate lawnmower	**1** 9 14	**2 3 6** 7 8 9 10 **11 12 13** 15 **17**	**2** 9 10 18
2	locate speaker	1 **10 12** 14	**1 3 4** **5** **6 7 8** 9 **10 11** **12** **13** 17	**1** 3 4 5 6 8 **9** 10 11 **18**
3	talker left or right	**1 2** 4 **5 8 9** 14	**1 2** 4 5 **6 8** 9 **10 11 12** **13 17**	**1 2 3** 4 **5 6** 7 **8** **9 10 11** 12 **13 16** 18
4	locate door slam	1 10 14	**2** 3 **5 6 8** 10 **11 12 13** 17	1 4 9 **16**
5	locate above/below	1 9 12	**2** 3 **4 6 7 8** 9 10 **11** 12 **13**	1 3 **9** 10 18
6	locate dog barking	1 10 12 14	**1 2 3 4 5 7 8** 9 **10 11** **12** **13 17**	1 4 8 **9** 10 18
7	locate vehicle	**1 5** 7 8 9 10 **12 14**	1 **2 5 6 8** **9** **10** **11** **12** **13**	2 4 7 9 **10** 1
8	distance voice/steps	**1** 2 4 5 6 7 8 9 10 **12** **14**	1 **2 3 4 5 6** **7** **9** **10** **11** **12** **13 17**	2 3 4 5 7 8 **9 10** 11 13 14 **18**
9	distance vehicle	**1** 3 5 8 9 **12 14**	1 2 3 5 6 **7** **8** **10** **11 12 13** 17	2 **9 10**
10	lateral movement vehicle	**1** 2 **5** 8 9 **10 12 14**	1 **2 3** 4 5 **6 7** **8** **9** **11** **12** **13**	1 **2** 3 4 5 7 9 10 **16**
11	lateral movement voice/steps	**1** 5 7 8 9 10 **12 14**	**1 2 3 4 5** **6** **7** **8** **9** **10** **12** **13 17**	**2** 3 4 5 6 7 8 **9** 10 11 **18**
12	voice/steps move to or from	**1** 2 5 9 **10** 12 **14**	**1 2** **3 4** 5 **6** **7** **8 9 10** **11** **13 17**	1 **2** 4 5 7 **8 9 10** 11 13 14 **18**
13	vehicle move to or from	**1 2** 3 **4** 7 8 **9 10 12 14**	**1 2** **3 4 5 6** **7** **8** **9** **10** **11** **12 17**	**1 2** 3 **4 5 6** 7 **8 9** **10** 11 **13** 14 18
14	internalize sounds			
15	sounds closer		1 16	
16	sounds further		15	
17	sounds where expect	1 2 9 **14**	**1** 2 **3** 4 **6 8** 9 **11 12 13**	**2** 4 **5** 7 **8 9 10 11** 12 **13 18**
	**III Other**			
1	sounds separate	1 2 4	**2 3** 4 5 6 10 12 **13**	**2 3** 4 **5 6 8** 9 **10** 11 **13**
2	sounds (not) jumbled	**1** 2 4 **5** 7 8 9 14	**1 3** 7 8 9 **10 11 12 13 17**	**1 3** 4 **5 6** 7 **8 9 10 11** 12 **13** 18
3	music and voice separate	1 4 5 6 9 14	2 **3** 5 8 10 11 13	**1 2 4** 5 6 7 **8** 9 **11 12 13 18**
4	identify by voice	**1 2** 14	2 3 4 6 7 8 10 11 12 **13** 17	1 2 **3 5 6** 7 **8 9** 10 **11** 12 **13 14** 17 18
5	distinguish music	1 **2** 5 9	2 **3** 8 10 11 12 **13 17**	**1 2** 3 **4 6** 7 **8 9 10 11 12 13**
6	distinguish sounds	1 2 5 14	**2** 3 11 **13**	**1 2 3 4** **5** 7 **8 9 10 11** 12 **13**
7	identify instruments	**5**	3 7 8 10 11 12 13 17	2 3 4 5 6 **8** 10 11 12 13
8	music natural	**1** 2 5 **8 9 14**	**2 3** 6 8 11 **12 13 17**	**1 2 3 4 5** **6** **7 9 10 11** **12** **13** 18
9	sounds clear	**1 2** 7 8 9 12 **14**	1 **2 3** 4 **5 6** 7 **8 9** 10 **11 12 13** **17**	1 **2** 3 **4 5 6 8 10 11 13 14** 18
10	voices natural	**1 2** 3 **4 5** 6 8 **9** 12 **14**	1 2 **3** 5 6 **7 8 9** 10 11 **12 13 17**	**1 2** 4 **5 6** 7 **8 9** **11** 12 **13** 18
11	sounds natural	1 2 5 9 14	2 **3** 7 8 11 12 13 **17**	**1 2 3 4** **5** **6** 7 **8 9 10** **12** **13**
12	own voice natural	9 14	3 17	2 **3** 4 **5** 6 7 **8** 10 **11 13**
13	tell mood from voice	**1 2** 3 4 5 9 14	**3** 8 12 **13 17**	**1 2 3 4** **5** **6** 7 **8 9 10 11 12** 14 17 18
14	concentrate when listen	**1** 2 10 14	8 12 13	**4 9** 13 **17 18** 19
16	understand when driver	**7** 8 **10** 11 12	**3 10**	
17	understand when passenger	14	4	13 **14** 18
18	effort of conversation	**1** 3 4 6 9 12 **14**	1 **2** 3 **4** 5 6 **8 11 12** 13 **17**	2 **3** 4 8 **9** 10 13 **14** 17 19
19	ignore competing sounds			14 18

Numbers in plain font show correlations of 0.50–0.59; bold type indicates correlations of 0.60–0.69; bold underlined type indicates correlations of >0.70.

**Table 4:** Partial correlations between SSQ items, controlling for better-ear HTL and worse-ear HTL; items correlating are identified by number within three subsections

Item	SSQ section	Items correlated, Speech	Items correlated, Spatial	Items correlated, Other
	**I Speech**
1	talk 1 TV on	2 4 14	1 2 3 4 8 10 11 **12 13**	1 2 3 **4** 6 **8** 9 **10 13** 14 **18**
2	talk 1 quiet	1 9	3 13	4 **5** 9 11 13
3	5 quiet see all	**4** 6 7 9 11 14		13 18
4	5 noise see all	1 **3** **6 7** 8 **9** 11 **12 14**		2 10 13 18
5	talk 1 noise	8 9	3 10	2 **7** 8
6	5 noise not see all	**3** **4 7** 9 11 12 14	8	18
7	talk where echoic	3 **4 6 8 9 11 12**		**16**
8	ignore voice same pitch	4 5 **7 9** **11 12** 14	3	8 16
9	ignore voice different pitch	2 3 **4** 5 6 **7 8** **11 12** 14	3 13	8 10
10	talk 1 *and* follow TV	12 14	2 7 **10** 12 13	**16**
11	talk 1 other voices	3 4 6 **7 8** **9** **12**		16
12	not miss speaker start	**4** 6 **7 8 9** 10 **11 14**	2 7 **8** 9 10 **11** 13	18
13	talk on phone		8 11	
14	follow 1 *and* phone	1 3 **4** 6 8 9 10 **12**	7 **8** 9 **10** 12 13 17	3 4 8 9 13 **18**
	**II Spatial**			
1	locate lawnmower	1	2 3 6 12 13 17	2
2	locate speaker	1 10 12	1 3 **4 5 6** 7 **8** 10 **11 12 13**	1 3 8 18
3	talker left or right	1 2 5 8 9	1 2 4 6 10 11 **12 13** 17	**1 2** 3 **5** 6 **8 9** 10 11 13 **16**
4	locate door slam	1	**2** 3 **5 6** 8 10 **11 12 13**	9 **18**
5	locate above/below		**2 4 6** 7 8 **11** 13	
6	locate dog barking		1 **2** 3 **4 5 8 11 12 13** 17	9
7	locate vehicle	10 12 14	2 5 **8** **9** **10** **11 12 13**
8	distance voice/steps	1 6 **12** 13 **14**	**2** 4 5 **6 7** **9 10 11 12 13** 17	4 9 14 18
9	distance vehicle	12 14	**7** **8 10 11 13**	2 16 11
10	lateral movement vehicle	1 5 **10** 12 **14**	2 3 4 **7 8 9 11 12 13**	2 **16** 11
11	lateral movement voice/steps	1 **12** 13	**2** 3 **4 5 6 7** **8 9 10** **12** **13** 17	2 3 9 18
12	voice/steps move to or from	**1** 10 14	1 **2 3 4 6 7 8 10 11 13** 17	8 9 18
13	vehicle move to or from	**1** 2 9 10 12 14	1 **2 3 4** 5** 6 7** **8 9 10** **11** **12 17**	1 2 3 **4** 5 6 8 **9 10** 11 13
14	internalize sounds			
15	sounds closer		16	
16	sounds further		15	
17	sounds where expect	14	1 3 6 8 11 12 **13**	2 5 8 **9** 10 11 **13** 18
	**III Other**			
1	sounds separate	1	2 **3** 13	**2** 3 **5 6** 8 9 10 13
2	sounds (not) jumbled	1 4 5	1 **3** 10 11 13 17	**1** 3 5 6 8 9 10 11 **13** 16
3	music and voice separate	1 14	2 3 11 13	1 2 **4** 5 6 **8** 11 12 13 18
4	identify by voice	**1** 2 14	8 **13**	**3 5 6** 8 9 10 **11 13 14** 18
5	distinguish music	**2**	**3** 13 17	**1** 2 3 **4 6** **8** 9 **10 11 12 13**
6	distinguish sounds	1	3 13	**1** 2 3 **4** **5** 7 **8** 9 10 **11** **13**
7	identify instruments	**5**		6 **8** 11
8	music natural	**1** 5 8 9 14	2 **3** 12 13 17	1 2 **3** 4 **5 6 7** 9 10 **11 12 13**18
9	sounds clear	1 2 14	**3** 4 6 8 11 12 **13 17**	1 2 4 5 6 8 10 11 13 **14 18**
10	voices natural	**1** 4 9	3 **13** 17	1 2 4 **5** 6 8 9 **11** 13
11	sounds natural	2	3 13 17	2 3 **4 5** **6** 7 **8** 9 **10 12 13**
12	own voice natural			3 **5 8 11 13**
13	tell mood from voice	**1** 2 3 4 14	3 13 **17**	1 **2** 3 **4** **5** **6** **8** 9 10 **11 12**
14	concentrate when listen	1	8	**4 9 17 18**
16	understand when driver	**7** 8 **10** 11	**3 10**	2
17	understand when passenger			**14**
18	effort of conversation	**1** 3 4 6 12 **14**	2 **4** 8 11 12 17	3 4 8 **9 14**
19	ignore competing sounds			

Numbers in plain font show partial correlations of 0.50–0.59; bold type indicates correlations of 0.60–0.69; bold underlined type indicates correlations >0.70.
