# Supplementary material for: The Speech, Spatial and Qualities of Hearing Scale (SSQ)
Source: Int J Audiol. Author manuscript; Available in PMC 2017 Sep 11. (PMC5593096; doi:10.1080/14992020400050014)
Supplement: SSQ B (pre/post) [file NIHMS73925-supplement-SSQ_B__pre_post_.pdf]

Speech Spatial Qualities -B

Advice about answering the questions

The following questions inquire about aspects of your ability and experience hearing and listening in different situations. You answered these questions previously, before you started wearing your hearing aid (or aids, if you use two hearing aids).

We would like you to answer the same questions, but comparing your ability and experience now, using your hearing aid/s, with your ability and experience before getting the aid/s.

For each question, put a mark, such as a cross (x), anywhere on the scale that runs from -5 through 0 to +5, shown against each question .

• If you are doing better now, with the hearing aid/s, in the situation described in a particular question, then put the mark somewhere to the right of the 0 point on the scale.

• If you are doing worse, then put the mark somewhere to the left of the 0.

• If things are no different, put the mark at around the 0 point.

• If things are *much* worse mark the scale at around -5; if things are *much* better mark it around +5.

We expect that all the questions are relevant to your everyday experience, but if a question describes a situation that does not apply to you, put a cross in the "not applicable" box. Please also write a note next to that question explaining why it does not apply in your case.

Please answer the following questions, then go on to the questions about your hearing

Your name :

Today's date :

Your age:

Please check one of these options:

I use **one** hearing aid (**left ear**) ☐

I use **one** hearing aid (**right ear**) ☐

I use **two** hearing aids (**both ears**) ☐

*If you have two aids and have used them for different lengths of time, please check the "**both ears"** box.*

How long have you been using a hearing aid or aids?

_____ years

_____ months

or

_____ weeks

1. *You are talking with one other person and there is a TV on in the same room. Without turning the TV down, can you follow what the person you're talking to says?*	**Comparing your ability now with your ability before getting your hearing aid/s**<br>*Much worse* *Unchanged* *Much better*<br>-5 -4 -3 -2 -1 0 +1 +2 +3 +4 +5<br>Not applicable ☐
2. *You are talking with one other person in a quiet, carpeted lounge-room. Can you follow what the other person says?*	**Comparing your ability now with your ability before getting your hearing aid/s**<br>*Much worse* *Unchanged* *Much better*<br>-5 -4 -3 -2 -1 0 +1 +2 +3 +4 +5<br>Not applicable ☐
3. *You are in a group of about five people, sitting round a table. It is an otherwise quiet place. You can see everyone else in the group. Can you follow the conversation?*	**Comparing your ability now with your ability before getting your hearing aid/s**<br>*Much worse* *Unchanged* *Much better*<br>-5 -4 -3 -2 -1 0 +1 +2 +3 +4 +5<br>Not applicable ☐
4. *You are in a group of about five people in a busy restaurant. You can see everyone else in the group. Can you follow the conversation?*	**Comparing your ability now with your ability before getting your hearing aid/s**<br>*Much worse* *Unchanged* *Much better*<br>-5 -4 -3 -2 -1 0 +1 +2 +3 +4 +5<br>Not applicable ☐
5. *You are talking with one other person. There is continuous background noise, such as a fan or running water. Can you follow what the person says?*	**Comparing your ability now with your ability before getting your hearing aid/s**<br>*Much worse* *Unchanged* *Much better*<br>-5 -4 -3 -2 -1 0 +1 +2 +3 +4 +5<br>Not applicable ☐

6. *You are in a group of about five people in a busy restaurant. You CANNOT see everyone else in the group. Can you follow the conversation?*	**Comparing your ability now with your ability before getting your hearing aid/s**<br>*Much worse* *Unchanged* *Much better*<br>-5 -4 -3 -2 -1 0 +1 +2 +3 +4 +5<br>Not applicable ☐
7. *You are talking to someone in a place where there are a lot of echoes, such as a church or railway terminus building. Can you follow what the other person says?*	**Comparing your ability now with your ability before getting your hearing aid/s**<br>*Much worse* *Unchanged* *Much better*<br>-5 -4 -3 -2 -1 0 +1 +2 +3 +4 +5<br>Not applicable ☐
8. *Can you have a conversation with someone when another person is speaking whose voice is the same pitch as the person you're talking to?*	**Comparing your ability now with your ability before getting your hearing aid/s**<br>*Much worse* *Unchanged* *Much better*<br>-5 -4 -3 -2 -1 0 +1 +2 +3 +4 +5<br>Not applicable ☐
9. *Can you have a conversation with someone when another person is speaking whose voice is different in pitch from the person you're talking to?*	**Comparing your ability now with your ability before getting your hearing aid/s**<br>*Much worse* *Unchanged* *Much better*<br>-5 -4 -3 -2 -1 0 +1 +2 +3 +4 +5<br>Not applicable ☐
10. *You are listening to someone talking to you, while at the same time trying to follow the news on TV. Can you follow what both people are saying?*	**Comparing your ability now with your ability before getting your hearing aid/s**<br>*Much worse* *Unchanged* *Much better*<br>-5 -4 -3 -2 -1 0 +1 +2 +3 +4 +5<br>Not applicable ☐

11. *You are in conversation with one person in a room where there are many other people talking. Can you follow what the person you are talking to is saying?*	**Comparing your ability now with your ability before getting your hearing aid/s**<br>*Much worse* *Unchanged* *Much better*<br>-5 -4 -3 -2 -1 0 +1 +2 +3 +4 +5<br>Not applicable ☐
12. *You are with a group and the conversation switches from one person to another. Can you easily follow the conversation without missing the start of what each new speaker is saying?*	**Comparing your ability now with your ability before getting your hearing aid/s**<br>*Much worse* *Unchanged* *Much better*<br>-5 -4 -3 -2 -1 0 +1 +2 +3 +4 +5<br>Not applicable ☐
13. *Can you easily have a conversation on the telephone?*	**Comparing your ability now with your ability before getting your hearing aid/s**<br>*Much worse* *Unchanged* *Much better*<br>-5 -4 -3 -2 -1 0 +1 +2 +3 +4 +5<br>Not applicable ☐
14. *You are listening to someone on the telephone and someone next to you starts talking. Can you follow what's being said by both speakers?*	**Comparing your ability now with your ability before getting your hearing aid/s**<br>*Much worse* *Unchanged* *Much better*<br>-5 -4 -3 -2 -1 0 +1 +2 +3 +4 +5<br>Not applicable ☐

Speech Spatial Qualities -B (Part 2: Spatial hearing)

1. *You are outdoors in an unfamiliar place. You hear someone using a lawnmower. You can't see where they are. Can you tell right away where the sound is coming from?*	**Comparing your ability now with your ability before getting your hearing aid/s**<br>*Much worse* *Unchanged* *Much better*<br>-5 -4 -3 -2 -1 0 +1 +2 +3 +4 +5<br>Not applicable ☐
2. *You are sitting around a table or at a meeting with several people. You can't see everyone. Can you tell where any person is as soon as they start speaking?*	**Comparing your ability now with your ability before getting your hearing aid/s**<br>*Much worse* *Unchanged* *Much better*<br>-5 -4 -3 -2 -1 0 +1 +2 +3 +4 +5<br>Not applicable ☐
3. *You are sitting in between two people. One of them starts to speak. Can you tell right away whether it is the person on your left or your right, without having to look?*	**Comparing your ability now with your ability before getting your hearing aid/s**<br>*Much worse* *Unchanged* *Much better*<br>-5 -4 -3 -2 -1 0 +1 +2 +3 +4 +5<br>Not applicable ☐
4. *You are in an unfamiliar house. It is quiet. You hear a door slam. Can you tell right away where that sound came from?*	**Comparing your ability now with your ability before getting your hearing aid/s**<br>*Much worse* *Unchanged* *Much better*<br>-5 -4 -3 -2 -1 0 +1 +2 +3 +4 +5<br>Not applicable ☐
5. *You are in the stairwell of a building with floors above and below you. You can hear sounds from another floor. Can you readily tell where the sound is coming from?*	**Comparing your ability now with your ability before getting your hearing aid/s**<br>*Much worse* *Unchanged* *Much better*<br>-5 -4 -3 -2 -1 0 +1 +2 +3 +4 +5<br>Not applicable ☐

6. *You are outside. A dog barks loudly. Can you tell immediately where it is, without having to look?*	**Comparing your ability now with your ability before getting your hearing aid/s**<br>*Much worse* *Unchanged* *Much better*<br>-5 -4 -3 -2 -1 0 +1 +2 +3 +4 +5<br>Not applicable ☐
7. *You are standing on the footpath of a busy street. Can you hear right away which direction a bus or truck is coming from before you see it?*	**Comparing your ability now with your ability before getting your hearing aid/s**<br>*Much worse* *Unchanged* *Much better*<br>-5 -4 -3 -2 -1 0 +1 +2 +3 +4 +5<br>Not applicable ☐
8. *In the street, can you tell how far away someone is, from the sound of their voice or footsteps?*	**Comparing your ability now with your ability before getting your hearing aid/s**<br>*Much worse* *Unchanged* *Much better*<br>-5 -4 -3 -2 -1 0 +1 +2 +3 +4 +5<br>Not applicable ☐
9. *Can you tell how far away a bus or a truck is, from the sound?*	**Comparing your ability now with your ability before getting your hearing aid/s**<br>*Much worse* *Unchanged* *Much better*<br>-5 -4 -3 -2 -1 0 +1 +2 +3 +4 +5<br>Not applicable ☐
10. *Can you tell from the sound which direction a bus or truck is moving, for example, from your left to your right or right to left?*	**Comparing your ability now with your ability before getting your hearing aid/s**<br>*Much worse* *Unchanged* *Much better*<br>-5 -4 -3 -2 -1 0 +1 +2 +3 +4 +5<br>Not applicable ☐

11. *Can you tell from the sound of their voice or footsteps which direction a person is moving, for example, from your left to your right or right to left?*	**Comparing your ability now with your ability before getting your hearing aid/s**<br>*Much worse* *Unchanged* *Much better*<br>-5 -4 -3 -2 -1 0 +1 +2 +3 +4 +5<br>Not applicable ☐
12. *Can you tell from their voice or footsteps whether the person is coming towards you or going away?*	**Comparing your ability now with your ability before getting your hearing aid/s**<br>*Much worse* *Unchanged* *Much better*<br>-5 -4 -3 -2 -1 0 +1 +2 +3 +4 +5<br>Not applicable ☐
13. *Can you tell from the sound whether a bus or truck is coming towards you or going away?*	**Comparing your ability now with your ability before getting your hearing aid/s**<br>*Much worse* *Unchanged* *Much better*<br>-5 -4 -3 -2 -1 0 +1 +2 +3 +4 +5<br>Not applicable ☐
14. *Do the sounds of things you are able to hear seem to be inside your head rather than out there in the world?*	**Comparing your experience now with your experience before getting your hearing aid/s**<br>*More inside head* *Unchanged* *More out there*<br>-5 -4 -3 -2 -1 0 +1 +2 +3 +4 +5<br>Not applicable ☐
15. *Do the sounds of people or things you hear, but cannot see at first, turn out to be closer than expected when you do see them?*	**Comparing your experience now with your experience before getting your hearing aid/s**<br>*Much closer* *Unchanged* *Less close*<br>-5 -4 -3 -2 -1 0 +1 +2 +3 +4 +5<br>Not applicable ☐

16. Do the sounds of people or things you hear, but cannot see at first, turn out to be further away than expected when you do see them?	**Comparing your experience now with your experience before getting your hearing aid/s**<br>*Much further* — *Unchanged* — *Less far*<br>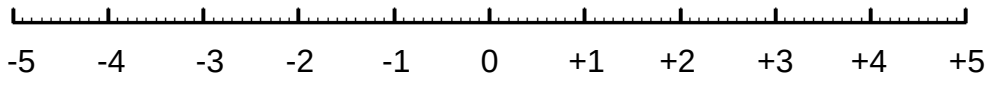 -5 -4 -3 -2 -1 0 +1 +2 +3 +4 +5<br>Not applicable ☐
17. Do you have the impression of sounds being exactly where you would expect them to be?	**Comparing your experience now with your experience before getting your hearing aid/s**<br>*Much worse impression* — *Unchanged* — *Much better impression*<br>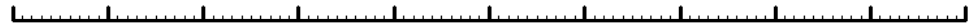 -5 -4 -3 -2 -1 0 +1 +2 +3 +4 +5<br>Not applicable ☐

Speech Spatial Qualities -B (Part 3: Qualities of hearing)

1. *Think of when you hear two things at once, for example, water running into a basin and, at the same time, a radio playing. Do you have the impression of these as sounding separate from each other?*	**Comparing your experience now with your experience before getting your hearing aid/s**<br>*Less separate* — *Unchanged* — *More separate*<br>-5 -4 -3 -2 -1 0 +1 +2 +3 +4 +5<br>Not applicable ☐
2. *When you hear more than one sound at a time, do you have the impression that it seems like a single jumbled sound?*	**Comparing your experience now with your experience before getting your hearing aid/s**<br>*More jumbled* — *Unchanged* — *Less jumbled*<br>-5 -4 -3 -2 -1 0 +1 +2 +3 +4 +5<br>Not applicable ☐
3. *You are in a room and there is music on the radio. Someone else in the room is talking. Can you hear the voice as something separate from the music?*	**Comparing your experience now with your experience before getting your hearing aid/s**<br>*Less separate* — *Unchanged* — *More separate*<br>-5 -4 -3 -2 -1 0 +1 +2 +3 +4 +5<br>Not applicable ☐
4. *Do you find it easy to recognise different people you know by the sound of each one's voice?*	**Comparing your ability now with your ability before getting your hearing aid/s**<br>*Much worse* — *Unchanged* — *Much better*<br>-5 -4 -3 -2 -1 0 +1 +2 +3 +4 +5<br>Not applicable ☐
5. *Do you find it easy to distinguish different pieces of music that you are familiar with?*	**Comparing your ability now with your ability before getting your hearing aid/s**<br>*Much worse* — *Unchanged* — *Much better*<br>-5 -4 -3 -2 -1 0 +1 +2 +3 +4 +5<br>Not applicable ☐

6. *Can you tell the difference between different sounds, for example, a car versus a bus; water boiling in a pot versus food cooking in a frypan?*	**Comparing your ability now with your ability before getting your hearing aid/s**<br>*Much worse* — *Unchanged* — *Much better*<br>-5 -4 -3 -2 -1 0 +1 +2 +3 +4 +5<br>Not applicable ☐
7. *When you listen to music, can you make out which instruments are playing?*	**Comparing your ability now with your ability before getting your hearing aid/s**<br>*Much worse* — *Unchanged* — *Much better*<br>-5 -4 -3 -2 -1 0 +1 +2 +3 +4 +5<br>Not applicable ☐
8. *When you listen to music, does it sound clear and natural?*	**Comparing your experience now with your experience before getting your hearing aid/s**<br>*Less clear and natural* — *Unchanged* — *More clear and natural*<br>-5 -4 -3 -2 -1 0 +1 +2 +3 +4 +5<br>Not applicable ☐
9. *Do everyday sounds that you can hear easily seem clear to you (not blurred)?*	**Comparing your experience now with your experience before getting your hearing aid/s**<br>*Less clear* — *Unchanged* — *More clear*<br>-5 -4 -3 -2 -1 0 +1 +2 +3 +4 +5<br>Not applicable ☐
10. *Do other people's voices sound clear and natural?*	**Comparing your experience now with your experience before getting your hearing aid/s**<br>*Less clear and natural* — *Unchanged* — *More clear and natural*<br>-5 -4 -3 -2 -1 0 +1 +2 +3 +4 +5<br>Not applicable ☐

11. *Do everyday sounds that you hear seem to have an artificial or unnatural quality?*	**Comparing your experience now with your experience before getting your hearing aid/s**<br>*Less natural* — *Unchanged* — *More natural*<br>-5 -4 -3 -2 -1 0 +1 +2 +3 +4 +5<br>Not applicable ☐
12. *Does your own voice sound natural to you?*	**Comparing your experience now with your experience before getting your hearing aid/s**<br>*Less natural* — *Unchanged* — *More natural*<br>-5 -4 -3 -2 -1 0 +1 +2 +3 +4 +5<br>Not applicable ☐
13. *Can you easily judge another person's mood from the sound of their voice?*	**Comparing your ability now with your ability before getting your hearing aid/s**<br>*Much worse* — *Unchanged* — *Much better*<br>-5 -4 -3 -2 -1 0 +1 +2 +3 +4 +5<br>Not applicable ☐
14. *Do you have to concentrate very much when listening to someone or something?*	**Comparing your experience now with your experience before getting your hearing aid/s**<br>*More need to concentrate* — *Unchanged* — *Less need to concentrate*<br>-5 -4 -3 -2 -1 0 +1 +2 +3 +4 +5<br>Not applicable ☐
15. *Do you have to put in a lot of effort to hear what is being said in conversation with others?*	**Comparing your experience now with your experience before getting your hearing aid/s**<br>*More effort* — *Unchanged* — *Less effort*<br>-5 -4 -3 -2 -1 0 +1 +2 +3 +4 +5<br>Not applicable ☐

16. When you are the driver in a car can you easily hear what someone is saying who is sitting alongside you?	**Comparing your ability now with your ability before getting your hearing aid/s**<br>*Much worse* *Unchanged* *Much better*<br>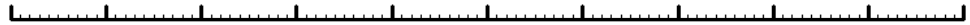 -5 -4 -3 -2 -1 0 +1 +2 +3 +4 +5<br>Not applicable ☐
17. When you are a passenger can you easily hear what the driver is saying sitting alongside you?	**Comparing your ability now with your ability before getting your hearing aid/s**<br>*Much worse* *Unchanged* *Much better*<br>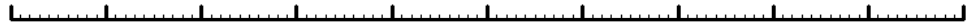 -5 -4 -3 -2 -1 0 +1 +2 +3 +4 +5<br>Not applicable ☐
18. Can you easily ignore other sounds when trying to listen to something?	**Comparing your ability now with your ability before getting your hearing aid/s**<br>*Much worse* *Unchanged* *Much better*<br>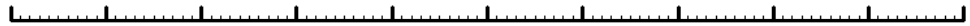 -5 -4 -3 -2 -1 0 +1 +2 +3 +4 +5<br>Not applicable ☐
