# Supplementary material for: The Speech, Spatial and Qualities of Hearing Scale (SSQ)
Source: Int J Audiol. Author manuscript; Available in PMC 2017 Sep 11. (PMC5593096; doi:10.1080/14992020400050014)
Supplement: SSQ original [file NIHMS73925-supplement-SSQ_original.pdf]

Speech Spatial Qualities

Advice about answering the questions

The following questions inquire about aspects of your ability and experience hearing and listening in different situations.

For each question, put a mark, such as a cross (x), anywhere on the scale shown against each question that runs from 0 through to 10. Putting a mark at 10 means that you would be perfectly able to do or experience what is described in the question. Putting a mark at 0 means you would be quite unable to do or experience what is described.

As an example, question 1 asks about having a conversation with someone while the TV is on at the same time. If you are well able to do this then put a mark up toward the right-hand end of the scale. If you could follow about half the conversation in this situation put the mark around the mid-point, and so on.

We expect that all the questions are relevant to your everyday experience, but if a question describes a situation that does not apply to you, put a cross in the "not applicable" box. Please also write a note next to that question explaining why it does not apply in your case.

Please answer the following questions, then go on to the questions about your hearing

Your name :

Today's date :

Your age:

Please check one of these options:

- I have **no** hearing aid/s ☐
- I use **one** hearing aid (**left ear**) ☐
- I use **one** hearing aid (**right ear**) ☐
- I use **two** hearing aids (**both ears**) ☐

If you have been using hearing aid/s, for how long?

______ years

______ months

or
_____ weeks

If you have two aids and have used them for <u>different</u> lengths of time, please write down both.

Speech Spatial Qualities (Part 1: Speech hearing)

1. You are talking with one other person and there is a TV on in the same room. Without turning the TV down, can you follow what the person you're talking to says?	*Not at all* 0 1 2 3 4 5 6 7 8 9 10 *Perfectly*	Not applicable ☐
2. You are talking with one other person in a quiet, carpeted lounge-room. Can you follow what the other person says?	*Not at all* 0 1 2 3 4 5 6 7 8 9 10 *Perfectly*	Not applicable ☐
3. You are in a group of about five people, sitting round a table. It is an otherwise quiet place. You can see everyone else in the group. Can you follow the conversation?	*Not at all* 0 1 2 3 4 5 6 7 8 9 10 *Perfectly*	Not applicable ☐
4. You are in a group of about five people in a busy restaurant. You can see everyone else in the group. Can you follow the conversation?	*Not at all* 0 1 2 3 4 5 6 7 8 9 10 *Perfectly*	Not applicable ☐
5. You are talking with one other person. There is continuous background noise, such as a fan or running water. Can you follow what the person says?	*Not at all* 0 1 2 3 4 5 6 7 8 9 10 *Perfectly*	Not applicable ☐

6. You are in a group of about five people in a busy restaurant. You CANNOT see everyone else in the group. Can you follow the conversation?	*Not at all* … *Perfectly*<br>0 1 2 3 4 5 6 7 8 9 10	Not applicable ☐
7. You are talking to someone in a place where there are a lot of echoes, such as a church or railway terminus building. Can you follow what the other person says?	*Not at all* … *Perfectly*<br>0 1 2 3 4 5 6 7 8 9 10	Not applicable ☐
8. Can you have a conversation with someone when another person is speaking whose voice is the same pitch as the person you're talking to?	*Not at all* … *Perfectly*<br>0 1 2 3 4 5 6 7 8 9 10	Not applicable ☐
9. Can you have a conversation with someone when another person is speaking whose voice is different in pitch from the person you're talking to?	*Not at all* … *Perfectly*<br>0 1 2 3 4 5 6 7 8 9 10	Not applicable ☐
10. You are listening to someone talking to you, while at the same time trying to follow the news on TV. Can you follow what both people are saying?	*Not at all* … *Perfectly*<br>0 1 2 3 4 5 6 7 8 9 10	Not applicable ☐

11. You are in conversation with one person in a room where there are many other people talking. Can you follow what the person you are talking to is saying?	*Not at all* … *Perfectly*<br>0 1 2 3 4 5 6 7 8 9 10	Not applicable ☐
12. You are with a group and the conversation switches from one person to another. Can you easily follow the conversation without missing the start of what each new speaker is saying?	*Not at all* … *Perfectly*<br>0 1 2 3 4 5 6 7 8 9 10	Not applicable ☐
13. Can you easily have a conversation on the telephone?	*Not at all* … *Perfectly*<br>0 1 2 3 4 5 6 7 8 9 10	Not applicable ☐
14. You are listening to someone on the telephone and someone next to you starts talking. Can you follow what's being said by both speakers?	*Not at all* … *Perfectly*<br>0 1 2 3 4 5 6 7 8 9 10	Not applicable ☐

Speech Spatial Qualities (Part 2: Spatial hearing)

1. You are outdoors in an unfamiliar place. You hear someone using a lawnmower. You can't see where they are. Can you tell right away where the sound is coming from?	*Not at all* … *Perfectly*<br>0 1 2 3 4 5 6 7 8 9 10	Not applicable ☐
2. You are sitting around a table or at a meeting with several people. You can't see everyone. Can you tell where any person is as soon as they start speaking?	*Not at all* … *Perfectly*<br>0 1 2 3 4 5 6 7 8 9 10	Not applicable ☐
3. You are sitting in between two people. One of them starts to speak. Can you tell right away whether it is the person on your left or your right, without having to look?	*Not at all* … *Perfectly*<br>0 1 2 3 4 5 6 7 8 9 10	Not applicable ☐
4. You are in an unfamiliar house. It is quiet. You hear a door slam. Can you tell right away where that sound came from?	*Not at all* … *Perfectly*<br>0 1 2 3 4 5 6 7 8 9 10	Not applicable ☐
5. You are in the stairwell of a building with floors above and below you. You can hear sounds from another floor. Can you readily tell where the sound is coming from?	*Not at all* … *Perfectly*<br>0 1 2 3 4 5 6 7 8 9 10	Not applicable ☐

6. You are outside. A dog barks loudly. Can you tell immediately where it is, without having to look?	*Not at all* … *Perfectly*<br>0 1 2 3 4 5 6 7 8 9 10	Not applicable ☐
7. You are standing on the footpath of a busy street. Can you hear right away which direction a bus or truck is coming from before you see it?	*Not at all* … *Perfectly*<br>0 1 2 3 4 5 6 7 8 9 10	Not applicable ☐
8. In the street, can you tell how far away someone is, from the sound of their voice or footsteps?	*Not at all* … *Perfectly*<br>0 1 2 3 4 5 6 7 8 9 10	Not applicable ☐
9. Can you tell how far away a bus or a truck is, from the sound?	*Not at all* … *Perfectly*<br>0 1 2 3 4 5 6 7 8 9 10	Not applicable ☐
10. Can you tell from the sound which direction a bus or truck is moving, for example, from your left to your right or right to left?	*Not at all* … *Perfectly*<br>0 1 2 3 4 5 6 7 8 9 10	Not applicable ☐

11. Can you tell from the sound of their voice or footsteps which direction a person is moving, for example, from your left to your right or right to left?	*Not at all* … *Perfectly*<br>0 1 2 3 4 5 6 7 8 9 10	Not applicable ☐
12. Can you tell from their voice or footsteps whether the person is coming towards you or going away?	*Not at all* … *Perfectly*<br>0 1 2 3 4 5 6 7 8 9 10	Not applicable ☐
13. Can you tell from the sound whether a bus or truck is coming towards you or going away?	*Not at all* … *Perfectly*<br>0 1 2 3 4 5 6 7 8 9 10	Not applicable ☐
14. Do the sounds of things you are able to hear seem to be inside your head rather than out there in the world?	*Inside my head* … *Out there*<br>0 1 2 3 4 5 6 7 8 9 10	Not applicable ☐
15. Do the sounds of people or things you hear, but cannot see at first, turn out to be closer than expected when you do see them?	*Much closer* … *Not closer*<br>0 1 2 3 4 5 6 7 8 9 10	Not applicable ☐

16. Do the sounds of people or things you hear, but cannot see at first, turn out to be further away than expected when you do see them?	*Much further* … *Not further*<br>0 1 2 3 4 5 6 7 8 9 10	Not applicable ☐
17. Do you have the impression of sounds being exactly where you would expect them to be?	*Not at all* … *Perfectly*<br>0 1 2 3 4 5 6 7 8 9 10	Not applicable ☐

Speech Spatial Qualities (Part 3: Qualities of hearing)

1. Think of when you hear two things at once, for example, water running into a basin and, at the same time, a radio playing. Do you have the impression of these as sounding separate from each other?	*Not at all* ... *Perfectly*<br>0 1 2 3 4 5 6 7 8 9 10	Not applicable ☐
2. When you hear more than one sound at a time, do you have the impression that it seems like a single jumbled sound?	*Jumbled* ... *Not jumbled*<br>0 1 2 3 4 5 6 7 8 9 10	Not applicable ☐
3. You are in a room and there is music on the radio. Someone else in the room is talking. Can you hear the voice as something separate from the music?	*Not at all* ... *Perfectly*<br>0 1 2 3 4 5 6 7 8 9 10	Not applicable ☐
4. Do you find it easy to recognise different people you know by the sound of each one's voice?	*Not at all* ... *Perfectly*<br>0 1 2 3 4 5 6 7 8 9 10	Not applicable ☐
5. Do you find it easy to distinguish different pieces of music that you are familiar with?	*Not at all* ... *Perfectly*<br>0 1 2 3 4 5 6 7 8 9 10	Not applicable ☐

6. Can you tell the difference between different sounds, for example, a car versus a bus; water boiling in a pot versus food cooking in a frypan?	*Not at all* ... *Perfectly*<br>0 1 2 3 4 5 6 7 8 9 10	Not applicable ☐
7. When you listen to music, can you make out which instruments are playing?	*Not at all* ... *Perfectly*<br>0 1 2 3 4 5 6 7 8 9 10	Not applicable ☐
8. When you listen to music, does it sound clear and natural?	*Not at all* ... *Perfectly*<br>0 1 2 3 4 5 6 7 8 9 10	Not applicable ☐
9. Do everyday sounds that you can hear easily seem clear to you (not blurred)?	*Not at all* ... *Perfectly*<br>0 1 2 3 4 5 6 7 8 9 10	Not applicable ☐
10. Do other people's voices sound clear and natural?	*Not at all* ... *Perfectly*<br>0 1 2 3 4 5 6 7 8 9 10	Not applicable ☐

Question	Scale	
11. Do everyday sounds that you hear seem to have an artificial or unnatural quality?	*Unnatural* — *Natural*<br>0 1 2 3 4 5 6 7 8 9 10	Not applicable ☐
12. Does your own voice sound natural to you?	*Not at all* — *Perfectly*<br>0 1 2 3 4 5 6 7 8 9 10	Not applicable ☐
13. Can you easily judge another person's mood from the sound of their voice?	*Not at all* — *Perfectly*<br>0 1 2 3 4 5 6 7 8 9 10	Not applicable ☐
14. Do you have to concentrate very much when listening to someone or something?	*Concentrate hard* — *Not need to concentrate*<br>0 1 2 3 4 5 6 7 8 9 10	Not applicable ☐
15. Do you have to put in a lot of effort to hear what is being said in conversation with others?	*Lots of effort* — *No effort*<br>0 1 2 3 4 5 6 7 8 9 10	Not applicable ☐

16. When you are the driver in a car can you easily hear what someone is saying who is sitting alongside you?	*Not at all* … *Perfectly*<br>0 1 2 3 4 5 6 7 8 9 10	Not applicable ☐
17. When you are a passenger can you easily hear what the driver is saying sitting alongside you?	*Not at all* … *Perfectly*<br>0 1 2 3 4 5 6 7 8 9 10	Not applicable ☐
18. Can you easily ignore other sounds when trying to listen to something?	*Not easily ignore* … *Easily ignore*<br>0 1 2 3 4 5 6 7 8 9 10	Not applicable ☐
