# Supplementary material for: The Speech, Spatial and Qualities of Hearing Scale (SSQ)
Source: Int J Audiol. Author manuscript; Available in PMC 2017 Sep 11. (PMC5593096; doi:10.1080/14992020400050014)
Supplement: Appendixes [file NIHMS73925-supplement-Appendixes.pdf]

Appendix 1. A sample item from the SSQ Questionnaire

1. You are talking with one other person and there is a TV on in the same room. Without turning the TV down, can you follow what the person you're talking to says?	Not at all Perfectly<br>0 1 2 3 4 5 6 7 8 9 10<br>Min Max	tick if not applicable ☐ or wouldn't hear it ☐

Appendix 2. Summary of the SSQ Items

SSQ Item	*Vignette*	*Anchors*
Speech 1	You are talking with one other person and there is a TV on in the same room. Without turning the TV down, can you follow what the person you're talking to says?	Not at all–Perfectly
Speech 2	You are talking with one other person in a quiet, carpeted lounge-room. Can you follow what the other person says?	Not at all–Perfectly
Speech 3	You are in a group of about five people, sitting round a table. It is an otherwise quiet place. You can see everyone else in the group. Can you follow the conversation?	Not at all–Perfectly
Speech 4	You are in a group of about five people in a busy restaurant. You can see everyone else in the group. Can you follow the conversation?	Not at all–Perfectly
Speech 5	You are talking with one other person. There is continuous background noise, such as a fan or running water. Can you follow what the person says?	Not at all–Perfectly
Speech 6	You are in a group of about five people in a busy restaurant. You *cannot* see everyone else in the group. Can you follow the conversation?	Not at all–Perfectly
Speech 7	You are talking to someone in a place where there are a lot of echoes, such as a church or railway terminus building. Can you follow what the other person says?	Not at all–Perfectly
Speech 8	Can you have a conversation with someone whose voice is the same pitch as that of the person you're talking with?	Not at all–Perfectly
Speech 9	Can you have a conversation with someone whose voice is a different pitch from that of the person you're talking with?	Not at all–Perfectly
Speech 10	You are listening to someone talking to you, while at the same time trying to follow the news on TV. Can you follow what both people are saying?	Not at all–Perfectly
Speech 11	You are in conversation with one person in a room where there are many other people talking. Can you follow what the person you are talking to is saying?	Not at all–Perfectly
Speech 12	You are with a group and the conversation switches from one person to another. Can you easily follow the conversation without missing the start of what each new speaker is saying?	Not at all–Perfectly
Speech 13	Can you easily have a conversation on the telephone?	Not at all–Perfectly
Speech 14	You are listening to someone on the telephone and someone next to you starts talking. Can you follow what's being said by both speakers?	Not at all–Perfectly

(*continued*)

Appendix 2 (*continued*)

SSQ Item	*Vignette*	*Anchors*
Spatial 1	You are outdoors in an unfamiliar place. You hear someone using a lawnmower. You can't see where they are. Can you tell right away where the sound is coming from?	Not at all–Perfectly
Spatial 2	You are sitting around a table or at a meeting with several people. You can't see everyone. Can you tell where any person is as soon as they start speaking?	Not at all–Perfectly
Spatial 3	You are sitting in between two people. One of them starts to speak. Can you tell right away whether it is the person on your left or your right, without having to look?	Not at all–Perfectly
Spatial 4	You are in an unfamiliar house. It is quiet. You hear a door slam. Can you tell right away where that sound came from?	Not at all–Perfectly
Spatial 5	You are in the stairwell of a building with floors above and below you. You can hear sounds from another floor. Can you readily tell where the sound is coming from?	Not at all–Perfectly
Spatial 6	You are outside. A dog barks loudly. Can you tell immediately where it is, without having to look?	Not at all–Perfectly
Spatial 7	You are standing on the footpath of a busy street. Can you hear right away which direction a bus or truck is coming from before you see it?	Not at all–Perfectly
Spatial 8	In the street, can you tell how far away someone is, from the sound of their voice or footsteps?	Not at all–Perfectly
Spatial 9	Can you tell how far away a bus or truck is, from the sound?	Not at all–Perfectly
Spatial 10	Can you tell from the sound which direction a bus or truck is moving, e.g. from your left to your right or right to left?	Not at all–Perfectly
Spatial 11	Can you tell from the sound of their voice or footsteps which direction a person is moving, e.g. from your left to your right or right to left?	Not at all–Perfectly
Spatial 12	Can you tell from their voice or footsteps whether the person is coming towards you or going away?	Not at all–Perfectly
Spatial 13	Can you tell from the sound whether a bus or truck is coming towards you or going away?	Not at all–Perfectly
Spatial 14	Do the sounds of things you are able to hear seem to be inside your head rather than out there in the world?	Inside my head–Out there
Spatial 15	Do the sounds of people or things you hear, but cannot see at first, turn out to be closer than expected when you do see them?	Much closer–Not closer
Spatial 16	Do the sounds of people or things you hear, but cannot see at first, turn out to be further away than expected when you do see them?	Much further–Not further
Spatial 17	Do you have the impression of sounds being exactly where you would expect them to be?	Not at all–Perfectly
Qualities 1	Think of when you hear two things at once, e.g. water running into a basin (a power tool being used) (a plane flying past) and, at the same time, a radio playing (the sound of hammering) (a truck driving past). Do you have the impression of these as sounding separate from each other?	Not at all–Perfectly
Qualities 2	When you hear more than one sound at a time, do you have the impression that it seems like a single jumbled sound?	Jumbled–Not jumbled
Qualities 3	You are in a room and there is music on the radio. Someone else in the room is talking. Can you hear the voice as something separate from the music?	Not at all–Perfectly

(*continued*)

Appendix 2 (*continued*)

SSQ Item	*Vignette*	*Anchors*
Qualities 4	Do you find it easy to recognize different people you know by the sound of each one's voice?	Not at all–Perfectly
Qualities 5	Do you find it easy to distinguish different pieces of music that you are familiar with?	Not at all–Perfectly
Qualities 6	Can you tell the difference between different sounds, e.g. a car versus a bus, or water boiling in a pot versus food cooking in a frying plan?	Not at all–Perfectly
Qualities 7	When you listen to music, can you make out which instruments are playing?	Not at all–Perfectly
Qualities 8	When you listen to music, does it sound clear and natural?	Not at all–Perfectly
Qualities 9	Do everyday sounds that you can hear easily seem clear to you (not blurred)?	Not at all–Perfectly
Qualities 10	Do other people's voices sound clear and natural?	Not at all–Perfectly
Qualities 11	Do everyday sounds that you hear seem to have an artificial or unnatural quality?	Unnatural–Natural
Qualities 12	Does your own voice sound natural to you?	Not at all–Perfectly
Qualities 13	Can you easily judge another person's mood from the sound of their voice?	Not at all–Perfectly
Qualities 14	Do you have to concentrate very much when listening to someone or something?	Concentrate Hard–Not need to concentrate
Qualities 15	If you turn one hearing aid/implant off, and do not adjust the other, does everything sound unnaturally quiet? (not relevant for unaided condition)	Too quiet–Not too quiet
Qualities 16	When you are the driver in a car, can you easily hear what someone is saying who is sitting alongside you?	Not at all–Perfectly
Qualities 17	When you are a passenger, can you easily hear what the driver is saying when sitting alongside you?	Not at all–Perfectly
Qualities 18	Do you have to put in a lot of effort to hear what is being said in conversation with others?	Lot of effort–No effort
Qualities 19	Can you easily ignore other sounds when trying to listen to something?	Not easily ignore–Easily ignore

Appendix 3: The 12 questions for the Handicap Questionnaire

1. How often does your hearing difficulty restrict the things you do?
2. How often do you feel worried or anxious because of your hearing difficulty?
3. As a result of your hearing difficulty, how often do you feel embarrassment when in the company of other people?
4. How often is your self-confidence affected by your hearing difficulty?
5. How often does your hearing difficulty make you feel nervous or uncomfortable?
6. How often does any difficulty with your hearing make you feel self-conscious?
7. How often does your difficulty with your hearing affect the way you feel about yourself?
8. How often are you inconvenienced by your hearing difficulty?
9. How often do you feel inclined to avoid social situations because of your hearing difficulty?
10. How often do you feel cut off from things because of your hearing difficulty?
11. How often does your hearing difficulty restrict your social or personal life?
12. How often do you feel tense and tired because of your hearing difficulty?

Response alternatives

(i) Never
(ii) Rarely
(iii) Sometimes
(iv) Often
(v) Almost always
